# Abnormal Condition Monitoring of Workpieces Based on RFID for Wisdom Manufacturing Workshops

**DOI:** 10.3390/s151229789

**Published:** 2015-12-03

**Authors:** Cunji Zhang, Xifan Yao, Jianming Zhang

**Affiliations:** 1 School of Mechanical and Automotive Engineering, South China University of Technology, Guangzhou 510640, China; mexfyao@scut.edu.cn (X.Y.); xtwgiqqk@163.com (J.Z.); 2 Department of Information Engineering, Guangxi College of Water Resources and Electric Power, Nanning 530023, China

**Keywords:** radio frequency identification (RFID), complex event processing (CEP), wisdom manufacturing, data cleaning, data mining

## Abstract

Radio Frequency Identification (RFID) technology has been widely used in many fields. However, previous studies have mainly focused on product life cycle tracking, and there are few studies on real-time status monitoring of workpieces in manufacturing workshops. In this paper, a wisdom manufacturing model is introduced, a sensing-aware environment for a wisdom manufacturing workshop is constructed, and RFID event models are defined. A synthetic data cleaning method is applied to clean the raw RFID data. The Complex Event Processing (CEP) technology is adopted to monitor abnormal conditions of workpieces in real time. The RFID data cleaning method and data mining technology are examined by simulation and physical experiments. The results show that the synthetic data cleaning method preprocesses data well. The CEP based on the Rifidi^®^ Edge Server technology completed abnormal condition monitoring of workpieces in real time. This paper reveals the importance of RFID spatial and temporal data analysis in real-time status monitoring of workpieces in wisdom manufacturing workshops.

## 1. Introduction

The monitoring and localization of objects have been an active research and development field in recent years. Existing techniques include Global Position System (GPS), infrared, Local Area Network (LAN) and ultrasound-based methods, *etc*. [1]. However, these technologies have some disadvantages. Thus, GPS technology is often used to locate outdoor objects, and is not suitable for indoor monitoring and localization due to its requirement of a direct line-of-sight communication to the satellites. Infrared methods also require a direct line-of-sight and involve a short-range signal transmission, and thus are not suitable for indoor monitoring and localization either. Wireless LAN technology is used to monitor and locate the objects by signal strength, and the target objects must be in the coverage area of wireless LAN, hence it might not be a good solution. Ultrasonic technology utilizes the Time of Flight (TOF) method to locate target objects. It usually requires either a transmitter or a receiver to accurately determine the location. Radio Frequency Identification (RFID) technology has become popular and been widely used in many fields due to its advantages [2], such as contactless communications, high data rate and security, no need for line-of-sight readability and low cost. With these advantages, RFID is a good candidate for workpiece monitoring and localization in manufacturing workshops.

RFID technology uses radio frequency waves to transfer data between readers and tagged objects, and provides fast data collection with precise identification of objects with unique IDs without line-of-sight, so it can be used for identifying, locating, tracking and monitoring physical objects [3]. With such significant technology advantages, RFID has been widely used for access control, objects tracking, smart box, highway tolls, logistics and supply chain, security and healthcare, *etc*. [4]. In particular, RFID has been adopted and deployed to collect various types of data in the manufacturing field [5].

With the integration and development of information technology and manufacturing technology, a number of advanced manufacturing models [6] have been proposed and applied, such as Lean Manufacturing (LM), Agile Manufacturing (AM), Virtual Manufacturing (VM), Virtual Enterprises (VE) and Intelligent Manufacturing (IM). Advanced manufacturing concerns not only manufacturing process, but also the full life cycle of market analysis, product design, manufacturing, assembly, sales, maintenance, services and recycle. Under the development and maturity of industrial wireless networks, Wireless Sensor Networks (WSN), RFID, Micro-Electrical Mechanical Systems (MEMS), Cyber-Physical Systems (CPS), and so on, the concept of Wisdom Manufacturing (WM) [7] is emerging.

As a service-oriented and knowledge-based humans-computers-things collaborative manufacturing model [8], WM puts emphasis on the fusion of the social, cyber and physical worlds, which form a Socio-Cyber-Physical System (SCPS). The main manufacturing resources consist of workers, machines, workpieces, *etc*. Manufacturing processes are prone to unexpected disturbances or disruptions such as workpiece anomaly, machine breakdown, urgent job arrival, lead time modification or changes in order quantities on short notice, the production of incorrect job quantities, materials or supplies failing to arrive when expected, misplaced parts, and tool defects [9]. Workpiece machining will be influenced by all these abnormal conditions in wisdom manufacturing workshops. Workers are obliged to respond to these disturbances in a timely manner, so as to reduce their impacts as much as possible. RFID technology has the potential to build an efficient real-time monitoring system.

**Figure 1:**
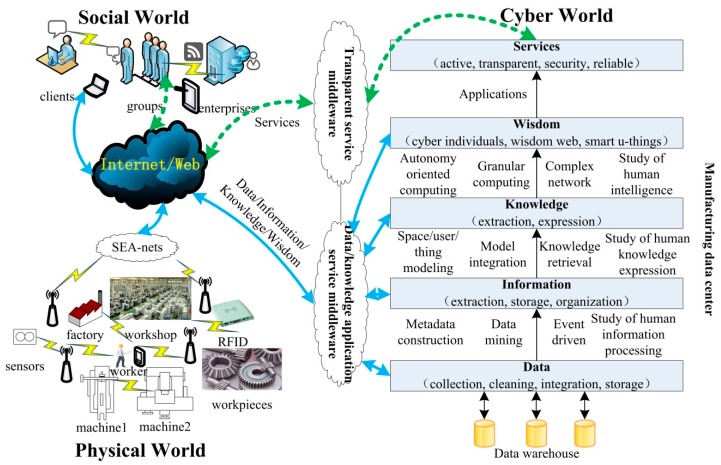
Wisdom manufacturing model based on SCPS [11].

Everything in the WM system can be aware of both itself and others to provide the right service for the right object at a right time and context [10]. The SCPS is the fusion of social, cyber and physical worlds. A WM model based on SCPS is illustrated in Figure 1 [11].

There are workshops, machines, workers, raw materials, workpieces, products, *etc*. in the physical world of wisdom manufacturing. These real physical things are called u-things if they are attached, embedded, or blended with computers, networks, and/or some other devices such as sensors, actuators, e-tags [12]. Through SEA-nets such as sensors net, embedded devices net and actuators net, manufacturing data such as the workshop environment data, machines state data, inventory data of raw materials and production data are transmitted to the web.

In the cyber world, the data level is involved with various data management and preprocessing technologies, including data collection, cleaning, integration and storage, *etc*. for completing the “things-data” conversion; data is converted into information through some methods such as metadata construction, data mining, event driven actions and the study of human information processing, The information level is involved with information extraction, information storage and information organization, *etc*. for completing the “data-information” conversion; Information is converted into knowledge through some methods such as space/user/thing modeling, model integration, knowledge retrieval and study of human knowledge expression. The knowledge level is involved with knowledge extraction and knowledge expression, *etc*. for completing the “information-knowledge” conversion; Knowledge is converted into wisdom through some methods such as autonomy-oriented computing, granular computing, complex network and study of human intelligence. The wisdom level is involved with cyber individuals, wisdom web and smart u-things, *etc*. for completing the “knowledge-wisdom” conversion; Wisdom is applied in various fields to provide active, transparent, security and reliable services. The service level is involved with services construction, services publishing and services integration for completing the “services-human” conversion.

In the social world, clients, social groups and enterprises implement effectively the implicit knowledge conversion and the integration of humans with the aid of social software (such as Blog, Tag, (Social Network Site) SNS and Wiki.). They use and enjoy a variety of products and services through the transparent service middleware and service platform, at the same time, and propose more personalized requirements for different products and services. These personalized requirements are converted into the product parameters to update the product gradually. Therefore, based on SCPS, the wisdom manufacturing model is formed.

**Figure 2:**
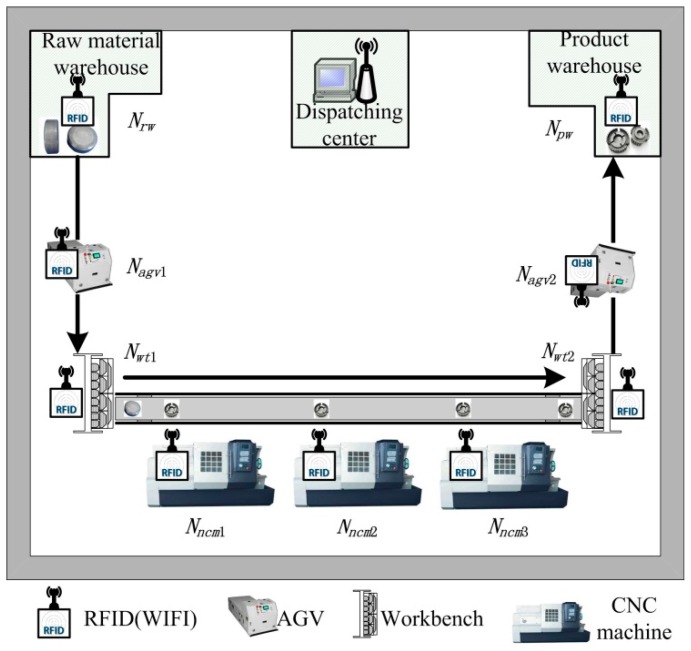
Sensing-aware environment of a wisdom manufacturing workshop.

In a wisdom manufacturing environment, the entire manufacturing workshop is covered by internet (such as LAN and WIFI). The sensing-aware environment of a wisdom manufacturing workshop is constructed based on cyber-physical systems, as shown in Figure 2. The manufacturing workshop contains dispatching center, raw material warehouse, (Automated Guided Vehicle) AGV, numerical control machines, workbenches, product warehouse, and so on. Directional (Ultra High Frequency) UHF RFID readers with LAN (WIFI) are installed on every workstation. Anti-metal ceramic RFID tags are pasted on every workpiece. The whole process from raw material to product is monitored. Each workstation is equipped with RFID sensing nodes, which sense ID numbers, time, location and other data of arrived workpieces in real time. Abnormal events can be monitored through data analysis in real time.

RFID applications in wisdom manufacturing are typically classified into two types [3]: (1) real-time-oriented monitoring applications; (2) history-oriented tracking applications. The fundamental characteristics of RFID data are temporal, dynamic, implicit semantic, inaccurate, integrated, flowing and large volume. Raw data provides no explicit semantic meaning for applications or business logics. It has to be transformed into semantic data properly represented with its own data models before it can be integrated into applications. Thus, RFID data model translates the physical world into its corresponding virtual (cyber) world. In this paper, passive tags are adopted, and RFID event processing models based on data mining are proposed to realize the monitoring of workpieces in wisdom manufacturing workshops.

The remainder of this paper is organized as follows: Section 2 reviews the literature of product monitoring, data cleaning and data mining based on RFID in manufacturing, and states the problems to be solved. Section 3 gives RFID event models consisting of tag event, simple event, and complex event. Section 4 proposes RFID data cleaning and data mining methods. Section 5 shows simulation and physical experiments to test the system functions. Conclusions and suggestions for future work are given in Section 6.

## 2. Literature Review

### 2.1. Monitoring Based on RFID in Manufacturing

RFID technology is widely applied throughout the manufacturing lifecycle, especially in materials or product management. In materials management, for example, an approach was presented to bridge the gap between the physical flow of materials on the shop floor and manufacturing information using RFID technology in discrete manufacturing environment [13]. In hyper environments, RFID was used to track and trace processes and supplies in construction and assembly industries, using networks of sensors/actuators and virtual reality [14]. A RFID-enabled real-time manufacturing information tracking infrastructure to address the real-time manufacturing data capturing and manufacturing information processing for extended enterprises [15] and traditional manufacturing resources such as employees, machines and materials equipped with RFID devices (readers and tags) to build the real-time data capturing environment was proposed. Qu *et al*. [16] proposed a RFID-based real-time shop floor materials management system, applying RFID for managing materials distribution in a complex assembly shop floor at a large air conditioner manufacturer. In product management, for example, a RFID-based intelligent decision support system architecture was proposed to handle production monitoring in a distributed clothing manufacturing environment [17], where RFID and cloud technologies were integrated for real-time and remote products monitoring. A Work In Progress (WIP) management framework based on smart objects such as RFID devices and web service technologies was proposed in a ubiquitous manufacturing [18]. Huang *et al*. [19] proposed an affordable approach to shop floor performance improvement by using wireless manufacturing with emphasis on how to deploy RFID technology for managing WIP inventories in manufacturing job shops with typical functional layouts; a refrigerated fruit storage monitoring system combining RFID and WSN was presented in [20], aiming to estimate energy consumption in a cold room, water loss from the products and detect any condensation over the stored commodities.

RFID technology has been increasingly applied to production planning and scheduling. For example, Zhong *et al.* [21] proposed a RFID-enabled real-time advanced production planning and scheduling shell to coordinate different decision makers across production processes for enhancing information sharing and coordinating decisions and operations of different parties involved in production planning, scheduling, execution and control. Qu *et al*. [22] discussed the item-level RFID implementation in terms of both real-time information control mechanism and system development environment, aiming to use RFID systems to enable the real-time coordination and interaction between the production planning and execution levels to achieve the lean control of manufacturing processes in smart assembly workshop. Dai *et al*. [23] proposed a RFID-enabled real-time manufacturing execution system at a typical (Small and Medium-sized Enterprise) SME engine valve manufacturer with the extension in setting up and integrating manufacturing execution system and enterprise resource planning system.

### 2.2. RFID Data Cleaning and Mining in Manufacturing

RFID raw data is inherently unreliable due to physical device limitations and different kinds of environmental noise. Kawakita *et al*. [24] discovered that the bit errors significantly degraded Class-1 Generation-2 protocol (C1G2) performance due to erroneous communication links. Buettner *et al.* [25] thought that physical effects such as errors and multipath degraded the overall performance of commercial readers. Therefore, RFID data cleaning is essential in order to correct the reading errors, aiming to allow these data streams to be used to make correct interpretations and analysis of the physical world they are representing.

A lot of approaches to clean RFID data have been studied in recent years with a focus on either fixed size sliding windows or adaptive sliding windows. For fixed size sliding windows, Bai *et al*. [26] proposed two types of filtering: false positive readings are removed from RFID data, and duplicate readings are merged into one distinct reading. The static size of the window is the limitation of this approach because a large window induces false positive readings and a small window cannot fill false negative readings. For adaptive sliding windows, Jeffery *et al*. [27,28] proposed the Extensible receptor Stream Processing (ESP) and Statistical sMoothing for Unreliable RFid data (SMURF) cleaning methods. ESP segments receptor stream processing into a cascade of five programmable stages: Point-Smooth-Merge-Arbitrate-Virtualize, which captures the context of temporal and spatial application layers by introducing the concept of temporal and spatial granularity. SMURF uses a statistical sampling-based approach to determine the“right” window size automatically and continuously adapts it over the life time of the system based on observed readings. This window size carefully balances two opposing application requirements: ensuring completeness for the set of tag readings (due to reader unreliability) and capturing tag dynamics (due to tag movements in and out of the reader’s detection field). Fan *et al*. [29] presented a behavior-based unreliable RFID data smoothing system to ensure a more complete access to get the movement behavior characteristics of tag. Massawe *et al*. [30] proposed an adaptive sliding window based approach called Window Sub-Range Transition Detection (WSTD), where a binomial sampling concept was used to calculate the appropriate window size and π-estimator to estimate the number of tags as proposed by SMURF. WSTD then used the comparison of the two window sub-range observations or estimated tag counts and some rules to detect when transition occurred within the window and then adjusted the window size appropriately. Li *et al*. [31] improved the SMURF algorithm by adding parameter p* and the reading rate to the reading cycle which was coming to the window. Zhao *et al*. [32] presented another confidence parameter η to decrease the false positive readings of original SMURF method based on the mathematical analysis. Most existing approaches for cleaning RFID data are rule-based inference algorithms with rather low accuracy. On the basis of above summary, a synthetic RFID data cleaning method based on SMURF is used to clean RFID tags in this paper.

The concepts and techniques of data mining were introduced to discover the invaluable new patterns from large number of data sets [33]. The volume of data generated by a RFID system is enormous due to redundancy and low level of abstraction, and the resulting main challenge then becomes how to handle and interpret the enormous volume of data in RFID applications [34]. In modern manufacturing, the volume of data grows at an unprecedented rate in the digital manufacturing environment. Such data may be related to design, products, machines, processes, materials, inventories, maintenance, planning and control, assembly, logistics, performance, *etc*. [35]. However, due to the “rich data but poor information” problem [36], data mining must be used on the collected manufacturing data, which contains valuable information and knowledge that could be integrated into the manufacturing system to improve decision making and enhance productivity [37].

As for RFID data mining, there exist many methods such as event model, Kriging method, and Procedure Tree. Of those methods, the event model is widely adopted. For example, an application framework for a real-time Complex Event Management System (CEMS) based on RFID device deployment was proposed in [38], which allowed users to obtain interested and meaningful information from large number of primitive events captured from the RFID devices in real time. An event-driven shop floor WIP management platform was created in Ubiquitous Manufacturing (UM) [39], aiming to monitor and control dynamic production and material handling through RFID-enabled traceability and visibility of shop floor manufacturing processes environment. A real-time Discrete Event (DE)-based monitoring system was developed for RFID-enabled shop floor monitoring in manufacturing [40], where the DE observer is designed to construct complex events from the simple events extracted from the raw RFID data. Huang *et al*. [41] studied abnormal event detection in the supply network, where the data captured from the (Electronic Product Code) EPC information service was used to calculate a path, and the machine learning method was adopted to cluster the path.

For the other RFID data mining methods, there also exist many examples that can be listed as follows: a schema-based RFID data storage model was presented to store and process RFID data efficiently in supply chain management systems [42], where a structure-based path splitting approach was proposed to intelligently and automatically split the movement paths of products. In tracking accuracy of the areas between the observed points, spatial and temporal analysis was applied to interpolate the continuous distribution of RFID tracking accuracy based on the Kriging method [43]. A holistic Big Data (BD) approach was proposed to extract frequent trajectory data from a massive collection of RFID-enabled shop floor logistics data [44], where enormous data could be collected and used for supporting further decision-makings such as logistics planning and scheduling; A method of configuring traditional manufacturing resources with RFID technology was proposed to capture and track real-time information during the manufacturing processes [45]. A data mining model was proposed to estimate the lead time from a real-life case [46], where the impact factors such as processing routine, batching strategy, scheduling rules and critical parameters were examined. A system architecture based on a data warehouse coupled with data mining functionalities was presented [47], which was capable of sending personalized offers to customers in the area of interest in real time. Masciari [48] introduced a Stream Monitoring enterprise Activities by RFID Tags (SMART) system based on an outlier template definition for detecting anomalies in RFID streams. Kwon *et al*. [49] proposed an advanced process management method, called “Procedure Tree” (PT), aiming to manage massive RFID data and perform real-time process management effectively. Kim *et al*. [50] suggested a method that applied RFID tag information and data mining technology to a manufacturing execution system (MES) for efficient process control in a TFT LCD production line.

From the above literature survey, we can see that abnormal condition monitoring of workpieces in manufacturing workshops is scarcely reported. As RFID applications in workpiece abnormal conditions are still in the starting stage, most research focuses on materials or product management.

### 2.3. Problem Statements

RFID technology has been widely used in manufacturing so far, and the application fields include product and process design, assembly, materials planning, quality control, scheduling, maintenance, *etc*. Most of literature studies focus on supply chain, objects tracking and product management, *etc*., while few researchers concentrate on abnormal condition monitoring of workpieces based on RFID. On the basis of the above summary, this paper proposes an abnormal condition monitoring method based on RFID in wisdom manufacturing workshops. The machining workpieces are monitored during the whole production process in real time, and abnormal condition of workpieces is mined on each workstation. This provides the basis of proactive scheduling and increases production efficiency in wisdom manufacturing workshops.

## 3. RFID Event Models

### 3.1. Event

The term event refers to the fact that something is happening (such as a change in the state of the system). An event is a happening of interest. In database applications, the interest in events comes mostly from the state changes that are produced by data manipulation [51]. In a monitored environment deployed with sensors, flows of observation data can be seen as streams of observable events. An event takes place, which refers to its occurrence, while an event is recognized by the system, which refers to its detection [52]. Event streams are sequences of event objects, which arrive in accordance with the order of the events. RFID events can be categorized as tag events, simple events (atomic events, basic events) and complex events (composite events, aggregated events) according to the particle size.

### 3.2. Tag Event

A tag event occurs when a RFID reader reads a tag. In other words, a tag is detected by a reader at a certain interval, a volume of fragmentary and redundancy tag events will occur in a short period of time. A tag event is denoted as *E_t_*, and a tag event model is defined as:

(1) $$E_{t}=e\left(w_{id},r_{id},t\right)$$

where *w_id_* is the Electronic Product Code (EPC) of a workpiece, *r_id_* is the Identification (ID) of a reader (the Internet Protocol (IP) address of a reader is bound with a workstation), and *t* is the time point of a tag event occurrence. There are a large number of unimportant and redundant events in collected tag events, which are filtered, accumulated, composited, reported and extracted into meaningful simple events.

### 3.3. Simple Event

A simple event is defined to occur at a certain time point or not occur at all. The simple event is used to directly represent the behavior of the system state. A simple event is denoted as *E_s_*, and a simple event model is defined as:

(2) $$E_{s}=e\left(w_{id},l,t\right)$$

where *w_id_* is the EPC of a workpiece, *l* is the location (workstation) of a workpiece when a simple event occurs, and *t* is the time point of a simple event occurrence. A simple event only reflects a single state of a workpiece at a time point, and simple events involved in each workstation are shown in Table 1. *AE* is the arrived event, and *LE* is the left (departed) event. *AE_sagv_*_1_ denotes the arrived event that a raw material or a workpiece (EPC is *w_id_*) arrives the workstation *N_agv_*_1_ at *t* time. *LE_srw_* denotes the left (departed) event that a raw material or a workpiece (EPC is *w_id_*) leaves the workstation *N_rw_* at *t* time. Other events denote the similar meanings, and each workstation location is shown in Figure 2.

**Table 1:** Simple events used in this paper.

Simple Event	Description	Events on All Workstations
Arrived Event: $AE_{s}=e\left(w_{id},l,t\right)$	A workpiece (EPC is *w_id_*) arrives the workstation *l* at *t* time.	*AE_sagv_*_1_, *AE_swt_*_1_, *AE_sncm_*_1_, *AE_sncm_*_2_, *AE_sncm_*_3_, *AE_swt_*_2_, *AE_sagv_*_2_, *AE_spw_*
Left Event: $LE_{s}=e\left(w_{id},l,t\right)$	A workpiece (EPC is *w_id_*) leaves the workstation *l* at *t* time.	*LE_srw_*, *LE_sagv_*_1_, *LE_swt_*_1_, *LE_sncm_*_1_, *LE_sncm_*_2_, *LE_sncm_*_3_, *LE_swt_*_2_, *LE_sagv_*_2_

### 3.4. Complex Event

A complex event is defined to apply to an event operator to constitute events that are simple events or other complex events [51]. A complex event is denoted as *E_c_*, and the complex event model is defined as:

(3) $$E_{c}=e\left(w_{id},l,e_{s},t_{s},t_{e}\right)$$

where *w_id_* is the EPC of a workpiece, *l* is the location (workstation) of a workpiece when a complex event occurs, *e_s_* is the sub-event set constituting a complex event, *t_s_* is the start time of a complex event occurrence, and *t_e_* is the end time of a complex event occurrence. If *t_s_* is equal to *t_e_*, the complex event occurs at a certain time point. Complex event operators used in this paper are shown in [53]. Complex events involved in each workstation are shown in Table 2. *SE* is the stayed event, and *DE* is the disappeared event. *SE_cagv_*_1_ denotes the stayed event that a raw material or a workpiece (EPC is *w_id_*) is located on the workstation *N_agv_*_1_ to stay or be machined from *t_s_* to *t_e_*. *DE_crw-agv_*_1_ denotes the disappeared event that a raw material or a workpiece (EPC is *w_id_*) is located on the dead zone temporally between the workstations *N_rw_* and *N_agv_*_1_ from *t_s_* to *t_e_*. Other events denote similar meanings.

**Table 2:** Complex events used in this paper.

Complex Event	Description	Events on All Workstations
Stayed (Machined) Event $SE_{c}=e\left(w_{id},l,e_{s},t_{s},t_{e}\right)$	A workpiece (EPC is *w_id_*) is located on the workstation *l* to stay or be machined from *t_s_* to *t_e_*.	*SE_cagv_*_1_, *SE_cwt_*_1_, *SE_cncm_*_1_, *SE_cncm_*_2_, *SE_cncm_*_3_, *SE_cwt_*_2_, *SE_cagv_*_2_
Disappeared Event $DE_{c}=e\left(w_{id},l^{\prime },e_{s},t_{s},t_{e}\right)$	A workpiece (EPC is *w_id_*) is located on the dead zone temporally between the workstations from *t_s_* to *t_e_*.	*DE_crw-agv_*_1_, *DE_cagv_*_1*-wt*1_, *DE_cwt_*_1*-ncm*1_, *DE_cncm_*_1*-ncm*2_, *DE_cncm_*_2*-ncm*3_, *DE_cncm_*_3*-*_*_swt_*_2_, *DE_cwt_*_2*-*_*_agv_*_2_, *DE_cagv_*_2*-*_*_pw_*

## 4. RFID Complex Event Processing

### 4.1. Complex Event Processing System

**Figure 3:**
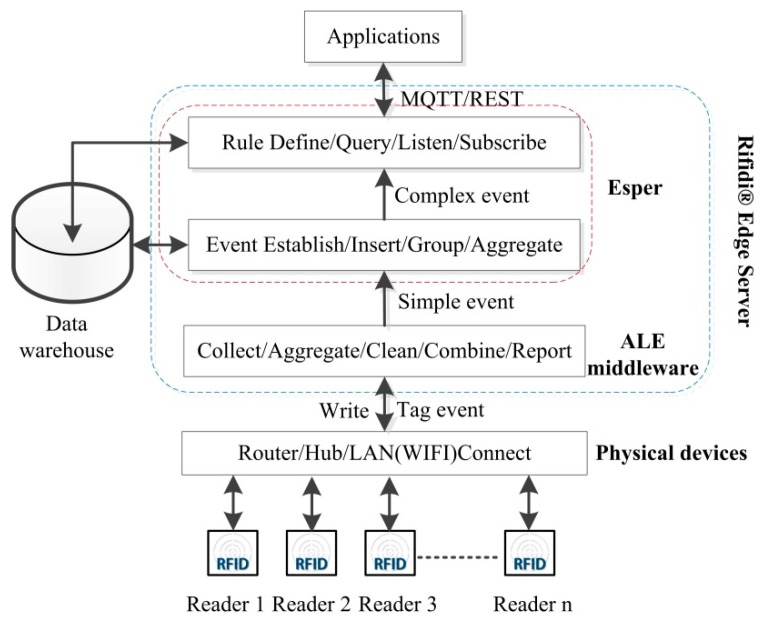
RFID complex event processing system.

RFID data is real-time and complex in logic. The workpiece condition information is mined from RFID data by CEP technology. The implementation of RFID CEP system is shown in Figure 3.

Readers with a LAN (WIFI) interface are located at different workstations. These readers are connected to the internet through the Router/Hub/LAN (WIFI), and tag data is transmitted via the internet in real time. Tag data collection, processing and release are implemented on the Rifidi^®^ Edge Server [54], which is an open source software from Transcends LLC (Glastonbury, CT, USA). It is a rapid development and configuration platform of RFID application system. This platform consists of four conceptual layers: sensor abstraction layer, application engine layer, communication layer (integration layer), and operations, administration & management layer. Diverse readers such as Impinj, Thinkify, ThingMagic, Alien and (Applied Wireless IDentifications group) AWID, are all supported by this platform, which contains (Application Level Events) ALE middleware and the Esper [53] complex event processing engine. A Derby database is embedded into this platform, which uses (Message Queue Telemetry Transport) MQTT information protocol and the Restlet plug-in to realize information transfer and operation management.

The tag data preprocessing is based on Application Level Events (ALE), and the ALE middleware is an international standard released by the EPC Global Organization for Standardization of EPC Processing Systems (such as reader.) and the interacting client. In terms of the RFID system, interactive activities include reading tags and writing tags. For reading tags, ALE collects RFID data and aggregates them at the same time interval. Reduplicative data is eliminated. Then, data is combined to reduce the amount of data. Finally, event reports are sent in different forms, and a simple event containing the intuitive information is formed.

Simple events are created (stated) and become semantic events, historical events in data warehouse are inserted, and these events are aggregated into complex events with the event operators. Complex events are processed by the Esper engine, founded by Bernhardt [55]. Esper is an open source complex event processing application platform, which contains a high performance event correlation (analysis) engine, and uses Event Processing Language (EPL) to define the client rules. The EPL syntax is found in [53]. Events are processed through Event Patterns and the Event Stream Queries (ESQ) methods, monitoring the occurrence of events and pushing the results to the subscribers.

### 4.2. RFID Data Cleaning

In a monitoring system based on RFID, readers communicate with tags by radio waves, and the system is quite vulnerable to environmental impacts. With the growing numbers of readers and tags, the interference will become particularly serious. These typical undesired scenarios caused by radio frequency interference include three aspects: false negative readings, false positive readings and duplicate readings [26].

False negative readings refer to that tags are in the vicinity of a reader but not detected by it. This can be due to two causes: (1) when multiple tags are to be simultaneously detected due to the interference of radio frequency collisions and signals, preventing the reader from identifying any tag; (2) a tag is not detected due to water or metal shielding or radiofrequency interference. False positive readings refer to that a tag is not present but captured. Besides RFID tags to be read, additional unexpected readings are generated. This can be caused by RFID tags outside the normal reading scope of a reader are captured by the reader. Duplicate readings refer to when tags are in the range of a reader for a long time and are read multiple times by the reader. It also occurs due to the tags in overlapping areas read by multiple readers. Data cleaning is key to abnormal condition monitoring using RFID data. The better data cleaning performs, the more accurate the abnormal detection results are.

#### 4.2.1. SMURF Method

SMURF is a method that dynamically adjusts the window size according to the average reading rate of the window based on a sliding window processor and binomial sampling theory. If the reading rate of a tag is lower, it sets a large window size to reduce false negative readings. On the other hand, if a tag reading rate is high, it sets a small window size to reduce false positive readings.

**Figure 4:**
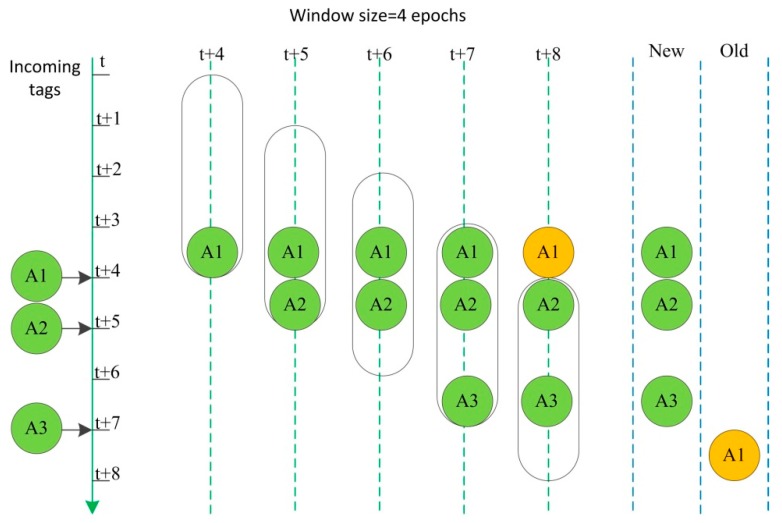
Sliding window theory.

The sliding window theory is illustrated in Figure 4. The window size is four epochs. Tag A1 enters the window at t + 4, which is thought as a new tag data. Tag A2 and A3 enter the window separately at t + 5 and t + 7. Meanwhile, Tag A1 remains in this window. Tag A1 leaves the sliding window at t + 8, and it is thought as an old tag data to be removed from the window.

The main idea is that the observed RFID readings can be viewed as a random sample of the population of tags in the physical world. Let *N_t_* denote the unknown size of the underlying tag population at epoch *t*, and let *S_t_* ⊆ {1,…, *N_t_*} denote the subset of tags observed (“sampled”) during that epoch. The key to this method is the use of a per-epoch sampling probability *p_i,t_* for each tag. Probability *p_i,t_* of tag *i* at epoch *t* can be calculated from the number of responses for the tag in combination with the known number of interrogation cycles per epoch using Equation (4):

(4) $$p_{i,t}=\frac{number\;of\;responses}{number\;of\;interrogation\;cycles}$$

The average read rate over all observation epochs is calculated from Equation (5):

(5) $$p_{i}^{avg}=\frac{\sum_{t\in S_{i}}p_{i,t}}{\left|S_{i}\right|}$$

where *S_i_* is the number of tag *i* sampled epochs in the sliding window.

At first, the problem of setting SMURF’s window size *w_i_* is considered to guarantee completeness with Equation (6):

(6) $$w_{i}\geq |\frac{\ln\left(1/\delta \right)}{p_{i}^{avg}}|$$

where *w_i_* is the number of epochs within the sliding window, δ is the required completeness confidence.

Equation (7) is a control condition to transit current size of sliding window and it can guarantee tag dynamics:

(7) $$\left|\left|S_{i}\right|-w_{i}p_{i}^{avg}\right|\geq 2\cdot \sqrt{w_{i}p_{i}^{avg}\left(1-p_{i}^{avg}\right)}$$

#### 4.2.2. Synthetic Method Based on SMURF

Li *et al*. [31] add a parameter p*, which is the reading rate of reading cycle about entering to the window, to determine whether to change the window size to the satisfaction of Equation (8):

(8) $$\omega <\left|\left(\frac{\sum_{n=1}^{n}p_{i}+p^{*}}{n+1}\right)-p^{*}\right|=p_{n}$$

where *ω* is the required probability threshold.

Zhao *et al*. [32] think that $p_{i}^{avg}$ is calculated simply by a mean method, but the mean value cannot measure how far a set of numbers is spread out or describe how far the numbers lie from the mean value. Variance *var_w_* is adopted to describe how the numbers of a data set change from Equation (9):

(9) $$var_{w}=\frac{\sum_{i=1}^{n}{\left(p_{i}-p_{i}^{avg}\right)}^{2}}{n}$$

A required tag dynamics confidence parameter *η* is adopted while $p_{i}^{avg}$ is calculated, and *var_w_* must satisfy Equation (10):

(10) $$var_{w}\leq \eta$$

A pseudo-code description of synthetic per-tag data cleaning algorithm is depicted in Algorithm 1. This method dynamically adjusts its window size for each tag, based on the guidance from its binomial-sampling model as discussed above with parameter δ, ω and η.

**Algorithm 1.** Synthetic per-tag data cleaning algorithm.Input: *T*=set of all observed tag IDs*δ*=required completeness confidence*ω*=required probability threshold*η*= required tag dynamics confidenceOutput: *t*= set of all present tag IDsInitialize: ∀*i*∈*T*, *w_i_*← 1**while** (g*etNextEpoch*) **do** **for** (*i* in *T*) **do**  *processWindow (W_i_)*→*p_i,t_*, $p_{i}^{avg}$, *p_n_*, *var_w_*, $\left|S_{i}\right|$  **if** (*ω<p_n_* ˄ *var_w_*≤*η*)**then**   $w_{i}^{*}$←*completeSize* ($p_{i}^{avg}$, *δ*)   **if** ($w_{i}^{*}$ > *w_i_*) **then**    *w_i_*←*max*{*min*{ *w_i_*+2, $w_{i}^{*}$},1}**else if** (*detectTransition* ($\left|S_{i}\right|$, *w_i_*, $p_{i}^{avg}$)) **then**    *w_i_*←*max*{*min*{*w_i_*/2, $w_{i}^{*}$},1}   **end if**  **end if**  **end for****end while**

### 4.3. Real-Time-Oriented Workpiece Monitoring Based on RFID Data Mining

RFID data is sequence data, streaming data and spatial-temporal data [33], which inflows into and outflows from a processing system at different update rates, and reflects the spatial-temporal characteristic of objects. The raw data provides no explicit semantic meaning, and has to be transformed into semantic data through an event model. Semantic data is further aggregated in different logic rules into semantic information indicating abnormal events. For this purpose, in this paper the complex event processing (streaming data processing) method is applied in RFID data mining to monitor workpiece abnormal conditions in manufacturing workshops. RFID data mining can be divided into real-time-oriented workpiece monitoring and history-oriented workpiece tracking. The former will be discussed in the following section.

In the manufacturing workshops, the IP address of a RFID reader is bound with a workstation (node), and the tag EPC is bound with a workpiece. The corresponding relationships of reader IPs, workstations and trigger events are shown in Table 3. The spatial-temporal and real-time RFID data is mined to monitor abnormal conditions of workpieces in real time, such as lack of raw materials, stayed (machined) time anomaly, workbench blocking and no product inventory. Estimating and detecting these abnormal conditions can make a preliminary decision according to the stayed (machined) time of a workpiece on every workstation, or whether a reader detects data. The results are shown in a state matrix, which provides a basis for further analysis and proactive scheduling.

**Table 3:** The corresponding relationships of reader IPs, workstations and trigger evens.

Reader IP	Workstation	Trigger Event
reader1 IP	*N_rw_*	*LE_srw_*
reader2 IP	*N_agv_*_1_	*AE_sagv_*_1_, *LE_sagv_*_1_, *SE_cagv_*_1_
reader3 IP	*N_wt_*_1_	*AE_swt_*_1_, *LE_swt_*_1_, *SE_cwt_*_1_
reader4 IP	*N_ncm_*_1_	*AE_sncm_*_1_, *LE_sncm_*_1_, *SE_cncm_*_1_
reader5 IP	*N_ncm_*_2_	*AE_sncm_*_2_, *LE_sncm_*_2_, *SE_cncm_*_2_
reader6 IP	*N_ncm_*_3_	*AE_sncm_*_3_, *LE_sncm_*_3_, *SE_cncm_*_3_
reader7 IP	*N_wt_*_2_	*AE_swt_*_2_, *LE_swt_*_2_, *SE_cwt_*_2_
reader8 IP	*N_agv_*_2_	*AE_sagv_*_2_, *LE_sagv_*_2_, *SE_cagv_*_2_
reader9 IP	*N_pw_*	*AE_spw_*

(1) Lack of Raw Materials and no Product Inventory

For workstation *N_rw_* equipped with a RFID reader in a raw materials warehouse, if data is not read for a long time, or event *LE_srw_* does not occur, an abnormal lack of raw materials condition occurs; For workstation *N_pw_* in a product warehouse, if data is not read for a long time, or event *AE_spw_* does not occur, an abnormal no product inventory condition occurs. Time threshold *t_rw-th_* and *t_pw-th_* are set, and the occurrence of event *LE_srw_* or *AE_spw_* in time threshold *t_rw-th_* or *t_pw-th_* is queried. For example, in a raw materials warehouse, the anomaly of raw materials lack is monitored by the following statement: Select * from pattern [every *LE_srw_*->timer:interval (*t_rw-th_*) and not *LE_srw_*];

(2) Stayed (Machined) Time Anomaly

For AGV workstations *N_agv_*_1_ and *N_agv_*_2_, machine workstations *N_ncm_*_1_, *N_ncm_*_2_ and *N_ncm_*_3_, assuming that the processing time is equal to the staying time on every workstation. The time threshold range is set with the lower threshold *t_low-th_*, and the higher threshold *t_high-th_*. Time attribute of stayed event *SE_cagv_*_1_, *SE_cncm_*_1_, *SE_cncm_*_2_, *SE_cncm_*_3_ or *SE_cagv_*_2_ is queried to judge whether the time attribute is within the scope of the threshold or not. If the threshold is exceeded, a stayed (machined) time anomaly occurs. For example, stayed (machined) event *SE_cncm_*_1_ is made up by simple events *AE_sncm_*_1_ and *LE_sncm_*_1_, the stayed (machined) time anomaly on workstation *N_ncm_*_1_ is monitored by the following statement:

Select time from *N_ncm_*_1_ (time in [*t_low-th_*: *t_high-th_*]);
Query the higher threshold:
Select *AE_sncm_*_1_ tag from pattern [every *AE_sncm1_* = *AE* -> timer:interval (*t_high-th_*) and not *LE_sncm_*_1_ (tag.tag.ID = *AE_sncm_*_1_ tag.tag.ID)];
Query the lower threshold:
Select *AE_sncm_*_1_ tag from pattern [every *AE_sncm_*_1_ = *AE* -> timer: interval (*t_low-th_*) and *LE_sncm_*_1_ (tag.tag.ID= *AE_sncm_*_1_ tag.tag.ID)];

(3) Workbench Blocking

The workbench is used to cache raw materials or products in workshops. If the cache time of raw materials or products is very long, the workbench will be blocked. For workbench workstation *N_wt_*_1_ and *N_wt_*_2_, time thresholds of caching raw materials and products are set by *t_wt_*_1*-th*_ and *t_wt_*_2*-th*_. Time attribute of stayed event *SE_cwt_*_1_ or *SE_cwt_*_2_ is queried to judge the time attribute within the scope of the threshold or not. If the threshold is exceeded, an abnormal workbench blocking condition occurs. For example, stayed event *SE_cwt_*_1_ is made up of simple events *AE_swt_*_1_ and *LE_swt_*_1_, the anomaly of workbench blocking on workstation *N_wt_*_1_ is monitored by the following statement:

Select *AE_swt_*_1_ tag from pattern [every *AE_swt_*_1_ = *AE*->timer:interval (*t_wt_*_1*-th*_) and not *LE_swt_*_1_ (tag.tag.ID= *AE_swt_*_1_.tag.tag.ID)];

Abnormal conditions of workpieces are monitored in real time through RFID data mining, which results in a real-time state matrix: $\text{S}=\left(a_{ij}\right)\left(1\leq i\leq m,1\leq j\leq 9\right)$, as shown in Equation (11), where *i* denotes a workpiece serial number, and *j* denotes a workstation serial number:

(11) $$\text{S}=(a_{ij})=[\begin{array}{ccccccccc} a_{11} & a_{12} & a_{13} & a_{14} & a_{15} & a_{16} & a_{17} & a_{18} & a_{19}\\ a_{21} & a_{22} & a_{23} & a_{24} & a_{25} & a_{26} & a_{27} & a_{28} & a_{29}\\ a_{31} & a_{32} & a_{33} & a_{34} & a_{35} & a_{36} & a_{37} & a_{38} & a_{39}\\ \vdots & \vdots & \vdots & \vdots & \vdots & \vdots & \vdots & \vdots & \vdots \\ a_{m1} & a_{m2} & a_{m3} & a_{m4} & a_{m5} & a_{m6} & a_{m7} & a_{m8} & a_{m9} \end{array}]$$

If $a_{ij}$ is equal to 1, the processed condition of the workstation is normal, whereas, If $a_{ij}$ is equal to 0, the processed condition of the workstation is abnormal. At the same time, all kinds of events are stored in the data warehouse as historical data for querying.

## 5. Experimental Results and Discussion

In this section, synthetic data cleaning and data mining techniques are evaluated experimentally. Three key points are illustrated: (1) the synthetic per-tag data cleaning algorithm is evaluated under the aid of MATLAB and a physical reader; (2) the simulation experiment of abnormal condition monitoring is tested by the Rifidi^®^ Edge Server; (3) abnormal condition monitoring is evaluated by physical experiments.

### 5.1. Synthetic Data Cleaning Experiment

In order to run experiments across a wide variety of scenarios, a physical reader and fifteen tags are set up to generate experimental data. The synthetic data cleaning algorithm is programmed in MATLAB. Two tag movement behaviors are investigated. The first is that a tag is static, which simulates how to place a tag suitably within the reader detection region, and the tag is monitored in a lower average errors per epoch. The second is that a tag is moved at a random initial velocity, which simulates dynamic environments such as tagged items on an AGV or conveyor belt, and the tag is monitored in a lower average errors per epoch at an appropriate velocity.

**Figure 5:**
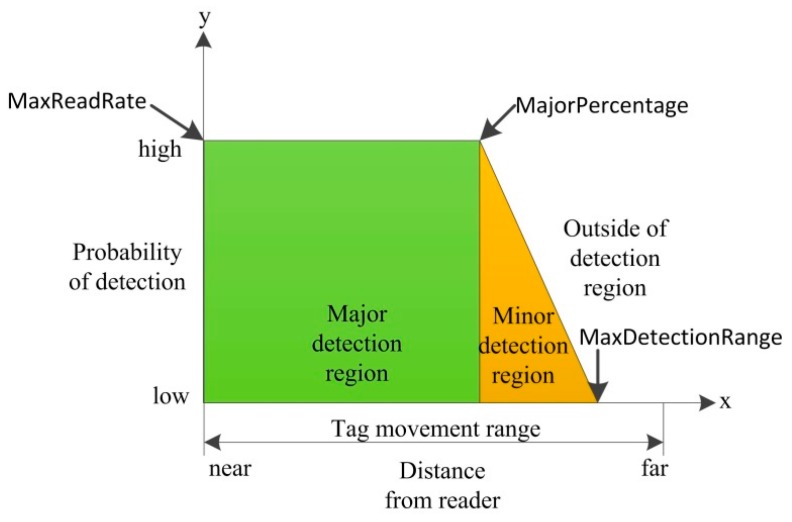
RFID tag-reader detection model.

The reader detection model is built based on the RFID tag-reader detection regions. Generally there are three distinct regions of operations of a passive RFID reader tag system: major detection region, minor detection region and outside of detection region [28]. The major detection region corresponds to roughly 75% of the full detection region, but it makes only 25% of the range in the noisy environment, as illustrated in Figure 5.

In the major detection region, the probability of detection maintains the max value (MaxReadRate). The tag read rate then degrades gradually with increasing distance to the minor detection region. In the outside of detection region, the read rate goes down to 0%. The main difference on this detection pattern when the tags are operated in different environments lies in the percentage of the reader’s detection range corresponding to its major detection region. The experimental parameters are summarized in Table 4.

**Table 4:** Experimental parameters.

Parameter (Device)	Value (Type)
*Reader*	MR6161E
*Tag*	MR6732
*TagMovementRange*	0–25 cm
*MaxDetectionRange*	20 cm
*MaxReadRate*	0.9
*MajorPercentage*	Varied
*NumTags*	15
*Velocity*	Varied
*epoch*	50 ms
*NumEpochs*	1000 epochs

The evaluation metric for per-tag cleaning is average errors per epoch. The average errors per epoch is calculated as $\sum_{i=1}^{NumEpochs}\left(FalsePositiveReadings_{i}+FalseNegativeReadings_{i}\right)/NumEpochs$. Based on Figure 5, a simplified reader detection model presented in [30] is adopted in Equation (12):

(12) $$p_{i,j}(x)=\{\begin{array}{cc} MaxReadRat, & x<MajorPercentage\\ \frac{MaxReadRate(MaxDetectionRange-x}{MaxDetectionRange-MajorPercentage}, & MajorPercentage\leq x\leq MaxDetectionRange\\ 0, & x>MaxDetectionRange \end{array}$$

Varying the *MajorPercentage* parameter simulates the reliability factors that affect the tag detection rates such as tag orientation and the radio frequency interference, while varying the distance (*x*) parameter simulates the tag-reader signal attenuation with distance. The *MajorPercentage* is varied between 0 and 100%. A lower value of *MajorPercentage* corresponds to a more unreliable environment and higher value of *MajorPercentage* corresponds to a more controlled environment.

**Figure 6:**
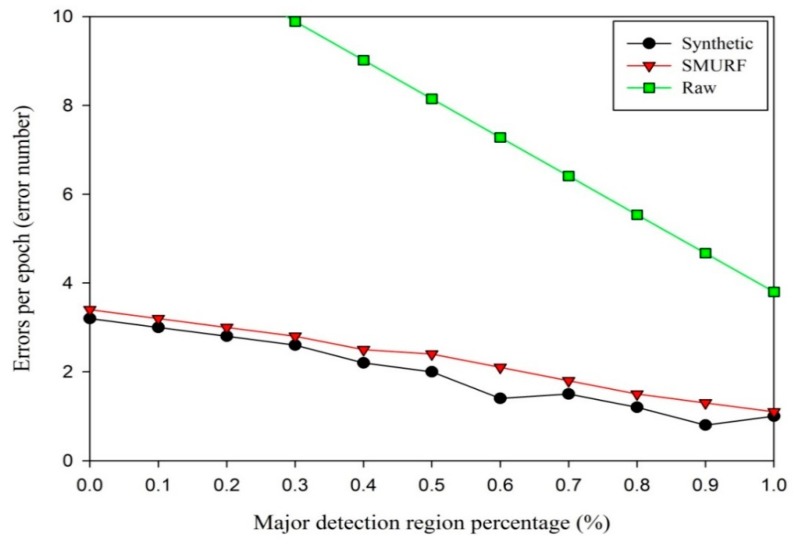
Average errors per epoch as *MajorPercentage* varies.

At first, the average errors per epoch is tested on the conditions of different levels of reader unreliability. For the static tag tests, the tag is static, but the *MajorPercentage* is varied. The results from data collected at every 1 cm within the range from 0 to 20 cm (the reader’s detection range is approximately 20 cm) are averaged. The repetitions of every data collection point are 20 times. The average errors per epoch is measured at each value for *MajorPercentage* between 0 and 1. Figure 6 shows the results of this experiment.

As can be seen from Figure 6, the synthetic data cleaning method results in lower errors than SMURF on the whole. The raw data straight line is truncated due to poor performance. As *MajorPercentage* increases, the accuracy of the two methods improves due to more reliable raw data. Especially, when the *MajorPercentage* is more than 0.6, the synthetic method works well. This region is adopted to monitor tagged workpieces in this paper. The difference of the synthetic method and SMURF method is evaluated by independent samples t-test [56]. The variance for the synthetic is 0.7162, and that for SMURF is 0.6176. The former is slightly bigger than the latter in static environment. The p-value of Levene’s test for equality of variances is 0.695 (Sig. = 0.695) assuming the variances of two methods are equal (*p* > 0.05). The p-value of t-test for equality of means is 0.385 (Sig. (2-tailed) = 0.385), and thus there is not a significant difference in the two data cleaning methods (*p* > 0.05).

**Figure 7:**
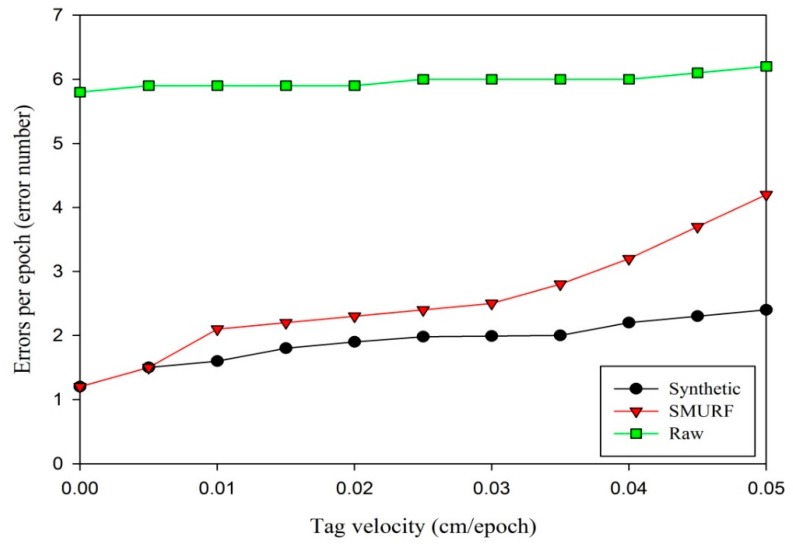
Average errors per epoch as tag velocity varies.

Next, the average errors per epoch is tested as the tag velocity varies. For the mobile tag tests, the *MajorPercentage* is fixed at 0.8 (representing a controlled environment). Meanwhile, the tag is moved back and forth between 0 and 25 cm from the reader at different velocities. The tag velocities are generated by a stepper motor. The results from data collected every 0.0025 cm/epoch from 0 to 0.05 cm/epoch are averaged. The repetitions of every data collection point are 20 times. The average errors per epoch is measured at each value for velocity from 0 to 0.05 cm/epoch. Figure 7 shows the results of this experiment.

In Figure 7, the synthetic data cleaning method performs better than SMURF. The average errors of raw data have little change as tag velocity varies. When the tag velocity is less than 0.01 cm/epoch, the two methods perform similarly. As tag velocity increases, the average errors of the two methods increase, but the synthetic data cleaning method shows lower average errors than SMURF. The difference of synthetic method and SMURF method is evaluated by an independent samples t-test. The variance for the synthetic approach is 0.1274, and that for SMURF is 0.7867. The former is smaller than the latter in a dynamic environment. The p-value of Levene’s test for equality of variances is 0.035 (Sig. = 0.035) assuming the variances of two methods are unequal (*p* < 0.05). The p-value of t-test for equality of means is 0.04 (Sig. (2-tailed) = 0.04), and thus there is a significant difference in the two data cleaning methods (*p* < 0.05).

In the above comparison results, the synthetic data cleaning method performs better than SMURF in two tag movement behaviors. In particular, there is a significant difference between the two cleaning methods in a dynamic environment. The key factor is whether the window size is calculated and adjusted precisely. The mechanism of adjusting the window size is different in the two methods. The SMURF approach adjusts the window size only based on the average reading rate of the window ($p_{i}^{avg}$) in Equations (6) and (7). For non-uniform RFID data stream, the SMURF performs poorer. However, the synthetic method considers the average reading rate ($p_{i}^{avg}$), the reading rate of reading cycle about entering to the window (p*) and Variance (*var_w_*) in Algorithm 1. Before calculating the window size, the condition (*ω* *<* *p_n_* ˄ *var_w_* ≤ *η*) must be satisfied. The parameter η is a required tag dynamics confidence in dynamic environment. The window size is calculated and adjusted precisely, the synthetic data cleaning method adopted in this paper shows much lower average errors in dynamic environment.

### 5.2. Abnormal Condition Monitoring Simulation Experiment

#### 5.2.1. Construction of Simulation Experiment Environment

A RFID complex event processing simulation environment is constructed by Eclipse and its plug-ins such as Rifidi SDK3.2 and Esper5.2 in a Windows 7 (32 bit) operating system environment. The virtual manufacturing system under the simulation environment is built according to the real environment layout of the wisdom manufacturing workshop in Figure 2. There are 10 workpieces, and the reader Alien is used and supported by the Rifidi^®^ Edge Server. The workpiece EPCs, reader IPs (the reader IP is replaced by a port number under a single PC simulation environment) and attribute threshold values are shown in Table 5.

**Table 5:** Workpiece EPCs, reader IPs and attribute threshold values.

Number	Workpiece EPC(*w_id_*)	Reader IP(*r_id_*)	Workstaion(l)	Attribute	Threshold (s)
1	35B2B5A08B3F39347F4A8FA7	127.0.0.1:10001	*N_rw_*	*t_rw-th_*	30
2	35ECDF34F4D15171B87B71AF	127.0.0.1:10002	*N_agv_*_1_	[*t_low-t_, t_high-th_*]	[40,45]
3	35877F002269B12EAF2198CF	127.0.0.1:10003	*N_wt_*_1_	*t_wt1-th_*	25
4	35B92D573F7956BAC1AD84F9	127.0.0.1:10004	*N_ncm_*_1_	[*t_low-th_,t_high-th_*]	[20,25]
5	35C3B9A3E13E8F32B9FE03A2	127.0.0.1:10005	*N_ncm_*_2_	[*t_low-t_, t_high-th_*]	[20,25]
6	3516BE6C0E9C8D4F8694992E	127.0.0.1:10006	*N_ncm_*_3_	[*t_low-th_, t_high-th_*]	[20,25]
7	358A1B77F25929686E907EE3	127.0.0.1:10007	*N_wt_*_2_	*t_wt2-th_*	25
8	3531F21B31DAD2058B9B2BC8	127.0.0.1:10008	*N_agv_*_2_	[*t_low-th_, t_high-th_*]	[40,45]
9	35417C666C5A2C7A2B635D8A	127.0.0.1:10009	*N_pw_*	*t_pw-th_*	30
10	355401990A44526D30609CE2				

#### 5.2.2. Results and Discussion of Simulation Experiment

**Figure 8:**
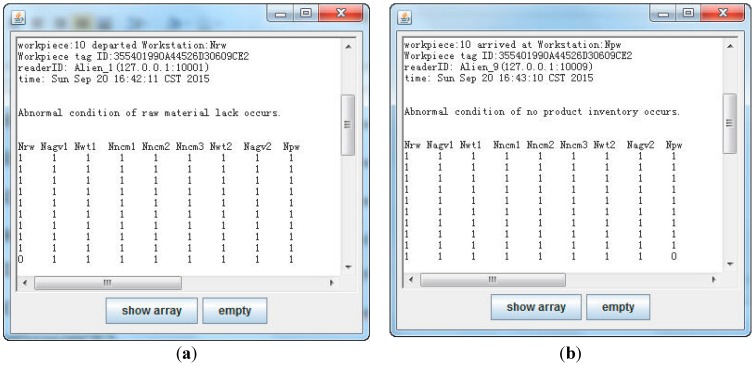

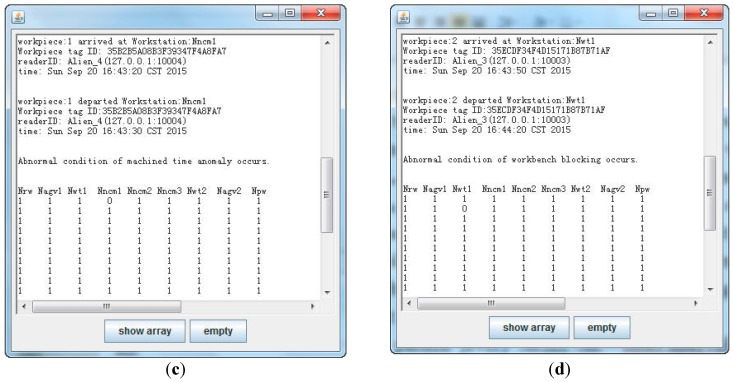
(**a**) Simulation result on workstation *N_rw_*; (**b**) Simulation result on workstation *N_pw_*; (**c**) Simulation result on workstation *N_ncm_*_1_; (**d**) Simulation result on workstation *N_wt_*_1_.

In this simulation experiment, it is assumed that the workpiece (*w_id_* = 355401990A44526D30609CE2) is moved from the raw materials warehouse, and afterwards, no other raw material is moved; The workpiece (*w_id_* = 355401990A44526D30609CE2) is transmitted into the product warehouse, and afterwards, no other product is transmitted; The workpiece (*w_id_* = 35B2B5A08B3F39347F4A8FA7) is processed on workstation *N_ncm_*_1_ for very short time (less than *t_low-th_*); The workpiece (*w_id_* = 35ECDF34F4D15171B87B71AF) stayed on workstation *N_wt_*_1_ for a long time (more than *t_wt1-th_*). For example, abnormal condition monitoring of raw material lack is depicted in Algorithm 2. The simulation result is shown in Figure 8.

**Algorithm 2.** Abnormal condition monitoring algorithm of raw material lack.StatementAwareUpdateListener NrwNoDepartedListener = **new** StatementAwareUpdateListener() **public**
**void** update (EventBean [] arg0, EventBean [] arg1, EPStatement arg2, EPServiceProvider arg3) **if** (arg0 != null)  System.out.printin ("Abnormal condition of raw material lack occurs."); addStatement ("select d.tag from pattern [every d=NrwDepartedEvent->timer:interval (30 sec) and not NrwDepartedEvent]", NrwNoDepartedListener);

### 5.3. Abnormal Condition Monitoring Physical Experiment

#### 5.3.1. Construction of Physical Experiment Environment

**Figure 9:**
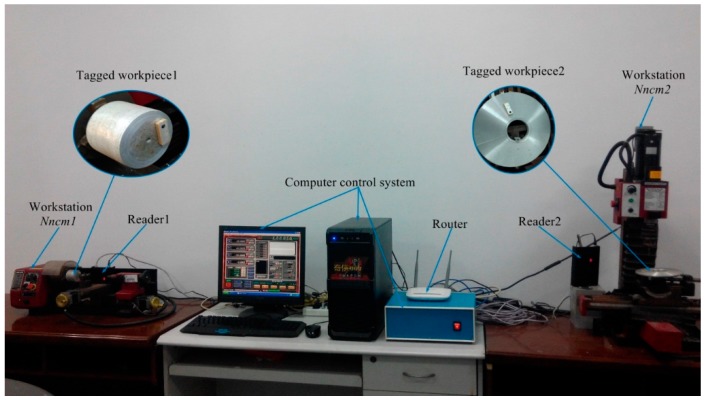
Physical experiment system of workpieces monitoring in real time.

In order to test the effectiveness of the proposed method, a physical experimental system for workpiece monitoring in real time is established and the experiments are carried out on the test rig. The architecture of the physical experiment system is shown in Figure 9. A mini-type CNC lathe is thought as the workstation *N_ncm_*_1_, a mini-type CNC milling machine is thought as the workstation *N_ncm_*_2_ A computer control system is used to control two CNC machines. The metal workpiece 1 and workpiece 2 with anti-metal ceramic tags are machined separately on workstation *N_ncm_*_1_ and *N_ncm_*_2_ Readers are placed on one side of every workstation to monitor the arrived and left (departed) time in real time. The real-time monitoring data is sent to the upper computer to process by the internet with routers.

The experimental parameters are summarized in Table 6. In the whole workpiece processing task, the tag is pasted on the non-machining surface of a workpiece, and the reader on every workstation monitors the arrived and left time of machined workpieces in real time. The upper computer collects the real-time data to clean and process these data with the CEP method. The machined time is judged whether anomalies occur or not.

**Table 6:** Parameters of physical experiment.

Number	Workpiece EPC (*w_id_*)	Reader IP (*r_id_*)	Workstation (*l*)	Attribute	Threshold (s)
1	300833B2DDD9014000000001	192.168.1.200	*N_ncm_*_1_	[*t_low-th_, t_high-th_*]	[20,25]
2	300833B2DDD9014000000002	192.168.1.201	*N_ncm_*_2_	[*t_low-th_, t_high-th_*]	[20,25]

#### 5.3.2. Results and Discussion of Physical Experiment

**Figure 10:**
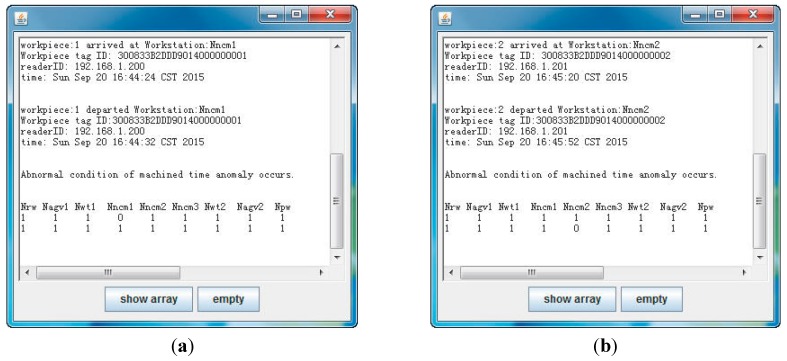
(**a**) Physical experiment result on workstation *N_ncm_*_1_; (**b**) Physical experiment result on workstation *N_ncm_*_2_.

There are two workstations in the physical experiment system, while there are nine in the simulation experiment. In the physical experiment, it is assumed that workpiece 1 (*w_id_* = 300833B2DDD9014000000001) is processed on workstation *N_ncm_*_1_ for less than 20 s; workpiece 2 (*w_id_*=300833B2DDD9014000000002) is processed on workstation *N_ncm_*_2_ for more than 25 s. The physical experiment result is shown in Figure 10.

The abnormal condition of workpiece 1 on workstation *N_ncm_*_1_ are shown in Figure 10a. The machined time was calculated based on the arrived time and left (departed) time, which was 8 s. The machined time was less than the threshold *t_low-th_* (20 s). As such, a machined time anomaly occurred, and *a*_14_ element in status matrix was 0. It was expressed that an abnormal condition of workpiece 1 occurred on workstation *N_ncm_*_1_. In Figure 10b, the machined time (32 s) was more than the threshold *t_high-th_* (25 s). Element *a*_25_ in status matrix was 0, which expressed that an abnormal condition of workpiece 2 occurred on workstation *N_ncm_*_2_.

## 6. Conclusions and Future Work

In this study, a RFID-based method of abnormal condition real-time monitoring of workpieces in wisdom manufacturing workshops is presented. RFID is used to detect the spatial-temporal information of workpieces at every workstation. Synthetic data cleaning and data mining based on CEP are applied to process RFID data in real time. The synthetic data cleaning method works better than SMURF in guaranteeing tag completeness and dynamics. Such real-time condition monitoring of workpieces provides a basis for proactive job shop scheduling in wisdom manufacturing.

However, this study only focuses on three abnormal conditions for real-time monitoring of workpieces in the wisdom manufacturing workshops. In the future work, more abnormal conditions such as urgent workpiece arrival, incorrect workpiece quantities and misplaced parts will be monitored by searching, aggregating and matching the EPCs of the workpieces, and the abnormal results being sent to the web. At the same time, we also plan to study the Prognostics and Health Management (PHM) of processing equipment in manufacturing workshops. For example, tools can be monitored and diagnosed with different sensors such as cutting force, vibration and acoustic emission, and deep learning is adopted to complete the tool condition monitoring and prognostics.
